# Notch1 Regulates Hippocampal Plasticity Through Interaction with the Reelin Pathway, Glutamatergic Transmission and CREB Signaling

**DOI:** 10.3389/fncel.2015.00447

**Published:** 2015-11-26

**Authors:** Emanuele Brai, Swananda Marathe, Simone Astori, Naila Ben Fredj, Elisabeth Perry, Christophe Lamy, Alessandra Scotti, Lavinia Alberi

**Affiliations:** ^1^Department of Medicine, Institute of Anatomy, University of FribourgFribourg, Switzerland; ^2^Department of Fundamental Neurosciences, University of LausanneLausanne, Switzerland; ^3^Histology Core Laboratory, Department of Cellular Biology and Anatomy, Georgia Health Sciences UniversityAugusta, GA, USA; ^4^Institute of Anatomy, Faculty of Medicine, University of BernBern, Switzerland; ^5^Unit of Pathology, Department of Medicine, University of FribourgFribourg, Switzerland

**Keywords:** Notch, ApoER2, NMDAR, CREB, plasticity, memory

## Abstract

Notch signaling plays a crucial role in adult brain function such as synaptic plasticity, memory and olfaction. Several reports suggest an involvement of this pathway in neurodegenerative dementia. Yet, to date, the mechanism underlying Notch activity in mature neurons remains unresolved. In this work, we investigate how Notch regulates synaptic potentiation and contributes to the establishment of memory in mice. We observe that Notch1 is a postsynaptic receptor with functional interactions with the Reelin receptor, apolipoprotein E receptor 2 (ApoER2) and the ionotropic receptor, N-methyl-D-aspartate receptor (NMDAR). Targeted loss of Notch1 in the hippocampal CA fields affects Reelin signaling by influencing Dab1 expression and impairs the synaptic potentiation achieved through Reelin stimulation. Further analysis indicates that loss of Notch1 affects the expression and composition of the NMDAR but not AMPAR. Glutamatergic signaling is further compromised through downregulation of CamKII and its secondary and tertiary messengers resulting in reduced cAMP response element-binding (CREB) signaling. Our results identify Notch1 as an important regulator of mechanisms involved in synaptic plasticity and memory formation. These findings emphasize the possible involvement of this signaling receptor in dementia.

**Highlights:**

In this paper, we propose a mechanism for Notch1-dependent plasticity that likely underlies the function of Notch1 in memory formation:

- Notch1 interacts with another important developmental pathway, the Reelin cascade.
- Notch1 regulates both NMDAR expression and composition.
- Notch1 influences a cascade of cellular events culminating in CREB activation.

## Introduction

Cell to cell communication pathways, which regulate the development and patterning of the mammalian brain have been shown to play an important role in neuronal network function and memory encoding in the adult brain (Herz and Chen, 2006; Oliva et al., 2013; Marathe and Alberi, 2015). In particular, the signaling receptors, which are the substrates of the γ-secretase complex, represent a synaptic hub (De Strooper and Annaert, 2001; Parks and Curtis, 2007) and are implicated in the memory deficit associated with Alzheimer’s disease (AD; Haass and De Strooper, 1999; De Strooper et al., 2012). Notch and Reelin signaling, which are both under γ-secretase’s regulation, functionally converge in mediating cortical migration and dendritic patterning (Gaiano, 2008; Hashimoto-Torii et al., 2008). Furthermore, Notch1 and Reelin loss of function mouse models display similar plasticity defects and memory impairments (Herz and Chen, 2006; Alberi et al., 2013; Trotter et al., 2013), suggesting that these cascades may also crosstalk at mature synapses.

Notch1 is a single-pass transmembrane receptor with an essential role in neural development (Gaiano and Fishell, 2002). Moreover, there is compelling evidence indicating that Notch1 has a critical role in adult brain function from invertebrates to mammals (Marathe and Alberi, 2015). Notch1 is expressed in the adult mammalian brain in neurons and stem cell niches (Stump et al., 2002). Activation of Notch1 occurs through binding to a specific ligand of the Delta Serrate ligand family, which is expressed on adjacent cells (Mumm et al., 2000). Upon ligand binding, the receptor can undergo sequential cleavage and generate the Notch intracellular domain (NICD; De Strooper et al., 1999), which has nuclear signaling function. NICD translocates to the nucleus and can bind to RBPJK inducing transcription of canonical targets (Kopan and Ilagan, 2009). Besides the canonical activity, there is mounting evidence that the Notch receptors display non-transcriptional functions, in a variety of cell types. The non-canonical signaling modality is achieved through the interaction with molecular players of other fundamental cellular cascades such as Abl, mTOR and Akt (Alberi et al., 2013). These crosstalks are just beginning to be understood and much less is known about Notch1 non-canonical signaling in neurons. Our own work has indicated that Notch1 processing is activity-dependent through the functional interaction with the early immediate gene Arc/Arg 3.1, which promotes γ-secretase’s activity (Alberi et al., 2011). Thus, sensory stimulation induces Notch activity in neuronal ensembles of the hippocampus and olfactory bulb (Alberi et al., 2011; Brai et al., 2014). As a result, Notch1 regulates synaptic plasticity and memory formation (Costa et al., 2003; Wang et al., 2004; Alberi et al., 2011). The mechanism underlying these functions has long been elusive and is the subject of the present study.

In this work, we show that Notch1 functionally interacts with other postsynaptic receptors such as Apolipoprotein E receptor 2 (ApoER2) and *N*-methyl-D-aspartate receptor (NMDAR) and influences their function. In particular, loss of Notch1 results in decreased glutamatergic transmission leading to diminished cAMP response element-binding (CREB) signaling. This mechanism may explain the synaptic deficit observed in the Notch1 loss of function mice and strongly supports the role of Notch1 at the synapse. Further studies will address whether Notch1 alterations contribute to the memory impairment in humans.

## Materials and Methods

### Animals

All experiments on mice were performed with permission of the local animal care committee (Canton of Fribourg, Switzerland) and according to the present Swiss law and the European Communities Council Directive of 24 November 1986 (86/609/EEC). Male mice were used in all experiments except for the LTP and whole-cell recordings. All animals (2–5 months of age, 30–35 g) were housed on 12 h light-dark cycle with access to food and water *ad libitum*. N1cKO and wild-type (WT) littermate mice (Notch1^flox/flox^ and CamKII::Cre (T29-1) were used in this study. Reeler heterozygous (Reln^+/−^) and WT (Reln^+/+^) littermate control were obtained from Dr. Knuesel, University of Zürich (Kocherhans et al., 2010).

### Novel Environmental Exploration

Wildtype and N1cKO mice were placed in a novel environment represented by a box (61 × 61 cm box with 24 cm with high walls containing visual cues and an open top) and let explore for 5 min (Alberi et al., 2011). After exploration, mice were returned to their cages and sacrificed 45 min later.

### Neuronal Cell Cultures

Neuronal cultures were prepared from the hippocampus of E17 WT embryos and plated on poly-L-lysine coated 60 mm dishes or 18 mm glass coverslips. Cultures were maintained in P-Neuronal media (PAA, Austria) supplemented with Neuromix (PAA, Austria) and 10% fetal bovine serum (FBS) as previously described (Banker and Cowan, 1977). After 14 days *in vitro*, neurons were fixed using 4% PFA and processed for fluorescent immunohistochemistry.

### Reelin Production

293T cells stably expressing recombinant Reelin were obtained from the laboratory of Dr. Curran (Benhayon et al., 2003). Cells were cultured in Dulbecco’s modified Eagle medium (DMEM; PAA, Austria) supplemented with 10% FBS (PAA, Austria), 10 U/ml penicillin/streptomycin (PAA, Austria) and 2mM L-glutamine (PAA, Austria) in presence of G418 (PAA, Austria) and Hygromicin B (Calbiochem). After cells were 70% confluent, the medium was changed and replaced with DMEM supplemented with penicillin/streptomycin and glutamine only. After 48 h the Reelin containing medium and the control medium were harvested. The supernatants were centrifuged at 12,000 rpm for 60 min, decanted and stored separately at −80°C until use. Efficient Reelin production was verified on small volumes (5; 10; 20 μl) of the harvested Reelin and compared to control media using electrophoresis and immunoblot analysis.

### Drugs

6-Cyano-7-nitroquinoxaline-2,3-dione (CNQX), D-(-)-2-Amino-5-phosphonopentanoic acid (D-AP5) and picrotoxin (PTX; Biotrend, Destin, FL, USA), N-(2,6-dimethylphenyl-carbamoyl-methyl)-triethylammonium-chloride (QX314) and glycine were from (Sigma-Aldrich, USA).

### Antibodies

The following antibodies were used for immunohistochemistry in this study: goat anti Notch1 (c-20; 1:500; sc-6014, Santa Cruz Biotechnology, USA), mouse anti NMDAR1 (NR1; 1:500; 556308, BD Pharmingen, UK), rabbit anti ApoER2 (1:500; ab108208, Abcam, UK), mouse anti Dab1 (1:500; gift of Dr. Scotti, UNIBE, Switzerland), rabbit anti Dab1 (1:500; MABS167, Millipore, Germany), rabbit anti Arc/Arg3.1 (1:500; gift of Prof. Worley, JHMI, USA). For immunohistochemistry on paraffin sections, goat anti Notch1 (c-20; 1:500; sc-6014, Santa Cruz Biotechnology, USA) and mouse anti NMDAR1 (1:500; NB300-118, Novus) were used. Following antibodies were used for immunoblot in this study: rabbit anti Notch1 (1:1000; 07-220, Upstate/Millipore, USA), mouse anti Notch1 (mN1A; 1:250; SAB4700742, Sigma Aldrich, USA), rabbit anti cleaved Notch1 (NICD; 1:500, #2421, Cell Signaling, USA), rabbit anti ApoER2 (1:500; ab108208, Abcam, UK), mouse anti Dab1 (1:500; gift of Dr. Scotti, UNIBE, Switzerland), rabbit anti phosphorylated Dab1 (1:500; ab126728, Abcam, UK), mouse anti NR1 (1:500; 556308, BD Pharmingen, UK), mouse anti NR2A (NR2A; 1:500; sc-390094, Santa Cruz Biotechnology, USA), mouse anti NR2B (NR2B; 1:500; sc-390094, Santa Cruz Biotechnology, USA), mouse anti GluR1 (1:500; sc-13152, Santa Cruz Biotechnology, USA), goat anti GluR2 (1:500; sc-7611, Santa Cruz Biotechnology, USA), mouse anti PSD95 (1:500; sc-32290, Santa Cruz Biotechnology, USA), mouse anti Reelin (1:500; G10, Merck, Germany), rabbit anti CamKII-α (1:1000; #3362, Cell Signaling, USA), rabbit anti phosphorylated ERK1/2 (pERK1/2; 1:1000; #9101, Cell Signaling, USA), mouse anti ERK1/2 (1:500; NBP 216703, Novus, UK), rabbit anti phosphorylated CREB (pCREB; 1:1000; #9198, Cell Signaling, USA), mouse anti CREB (1:1000; NB100-74393, Novus, UK), mouse anti GAPDH (1:5000; 1D4, Novus, UK), mouse anti β-Actin (1:2000; sc-8432, Santa Cruz Biotechnology, USA).

The following antibodies were used for immunoprecipitation: rabbit anti Notch1 (10 μg/1.2 mg of lysate; 07-220, Upstate/Millipore, USA), mouse anti ApoER2 (7 μg/1.2 mg of lysate; gift of Dr. Nimpf, Max Perutz Laboratories, Vienna, Austria), mouse anti Dab1 (10 μg/1.2 mg of lysate; gift of Dr. Nimpf, Max Perutz Laboratories, Vienna, Austria) and mouse anti NMDAR1 (10 μg/1.2 mg of lysate; 556308, BD Pharmingen, UK).

The secondary antibodies used in the study for immunohistochemistry were directly conjugated to Cy2, Cy3 or Cy5 and were raised in donkey (all 1:500; Jackson Immuno Europe, UK). After the completion of immunofluorescence protocols, neurons on glass coverslip were stained with phalloidin (1:1000; ab176753, Abcam, UK) to visualize F-actin puncta. Glass coverslips and sections were stained with DAPI (0.1mg/ml; 10236276001 Roche, Sigma-Aldrich, USA) to visualize nuclear morphology. Sections were mounted on Super frost slides (Thermofisher, USA). Glass coverslips and slides were mounted and coverslipped respectively using a custom made Polyvinyl alchool (PVA) and 1,4-diazabicyclo[2.2.2]octane (DABCO)-based mounting medium.

### Immunofluorescence

Mice were sacrificed by transcardial perfusion with 0.9% saline solution followed by a solution of 4% PFA. The brains were post-fixed overnight in 4% PFA and subsequently cryoprotected for two overnights in 30% sucrose solution. Fifty micrometer thick coronal sections were cut on a cryostat (Leica, Germany) in the anterior-posterior plane: −1.55 to −2.355 from Bregma. The sections were stored in PBS with 0.01% Sodium azide (Sigma-Aldrich, Germany) at 4°C until further analysis. For the intensity correlation analysis on CA fields, brains were embedded in paraffin. Six micrometer sections were cut in the coronal plane region comprising −1.96 and −2.06 from Bregma. The immunohistochemistry stainings using fluorescently tagged secondary antibodies from *in vitro* cultures and mouse brain sections were done as previously described (Alberi et al., 2011; Brai et al., 2014). Specimens were imaged using a Leica TCS SP5 confocal microscope (Leica Germany) with 40× and 63× objectives. All confocal images were calibrated on secondary control immunolabelled primary neurons and brain sections (Supplementary Figure 1).

### Immunoelectronmicroscopy (IEM)

Mouse brains were perfused with an IEM fixative buffer (4% paraformaldehyde, 0.2% gluaraldehyde in 0.1M cacodylate buffer). Brains were vibratomed coronally through the hippocampus and stored in IEM fixative until beginning the experiment. Vibratomed sections were put in permeabilization solution for 1 h and 30 min. Slices were washed thoroughly with Hepes Buffered Saline (HBS) and permeabilized with HBS plus 1% BSA and 0.0025% Triton X-100. Notch1 antibody (1:500; sc-6014, Santa Cruz Biotechnology, USA) was added at a dilution of 1:750 and incubated overnight on a shaker at 4°C. The next day, the sections were washed three times in HBS-0.05% BSA and then incubated in anti-species specific nanogold-conjugated antibody diluted 1:250 at 4°C overnight on a shaker. Slices were then washed three times in HBS-0.05% BSA for 5 min and washed with four changes of distilled water for 2 h. Slices were placed in 0.5 ml of Goldenhance EM^TM^ mixed according to manufacturer’s directions and incubated on a shaker for 2 h. Slices were washed thoroughly in ice-cold water to stop the gold enhancement and rinsed twice in HBS for 5 min. Slices were then washed in 0.1M cacodylate buffer, dissected to include the CA1 apical layer and thereafter embedded. Slices were post-fixed in 1% OsO_4_ plus 1.5% potassium ferrocyanide in cacodylate buffer for 1 h and post fixed in 1% OsO_4_ in cacodylate buffer. Sections were stained in 2% aqueous uranyl acetate on a shaker at room temperature for 1 h. After dehydration in an ascending ethanol series (50, 70, 90 and 100%), slices were placed in 1:1 mixture of propylene oxide/Embed 812 resin mixture for 1 h, then put in 100% Embed 812 resin mixture overnight on a rotator. Slices were flat embedded and polymerized at 60°C for 24 h. Thin sections were cut with a diamond knife on a Leica EM UC6 ultramicrotome (Leica Microsystems, Germany), collected on copper grids and stained with lead citrate. Sections were observed in a JEM 1230 transmission electron microscope (JEOL USA Inc., Peabody, MA, USA) at 110 kV and imaged with an UltraScan 4000 CCD camera & First Light Digital Camera Controller (Gatan Inc., Pleasanton, CA, USA).

### Transcript Expression Analysis by qPCR

Mice were transcardially perfused with 0.9% saline solution. The brain was dissected out and was transferred into an ice-cold Phosphate buffered saline (PBS) solution. The hippocampus was dissected out and the CA region was obtained by gently peeling the DG apart under a dissection microscope (Nikon, Japan). The tissue was flash-frozen in liquid nitrogen and stored at −80°C until further use. Total RNA was extracted using peqGOLD TriFast reagent (Peqlab, Germany) from isolated CA fields. Total RNA was quantified and the quality was assessed with a Nanodrop (NanoDrop2000, Thermo Scientific). Two micrograms of RNA per sample were subjected to reverse transcription using M-MLV Reverse Transcriptase (Promega, USA). Gene expression analysis was done by RT-qPCR (GoTaq^®^ qPCR Master Mix, Promega, USA) using gene specific primers (Table 1) with a Rotorgene (Qiagen, Germany). Quantitative PCR data analysis was performed using the ΔΔCt method as previously described (Bookout and Mangelsdorf, 2003). Gene expression analysis data were normalized to the endogenous housekeeping gene, β-actin.

**Table 1:** **qPCR primers sequences**.

Gene	Forward primer	Reverse primer
*β-actin*	GTG ACG TTG ACA TCC GTA AAG A	GCC GGA CTC ATC GTA CTC C
*Notch1*	TCA GAG GCC AGC AAG AAG AA	GCT CCT CAA ACC GGA ACT TC
*Reelin*	TTA CTC GCA CCT TGC TGA AAT	CAG TTG CTG GTA GGA GTC AAA G
*ApoER2*	TCC TGC CGA GAA GTT AAG CTG	AAG AAC GCA AGT CCC ATC CC
*Dab1*	GTG CTG TGA CCC AAT TAG AAC T	GAC GGG AGA AAG GCA TCA CC
*Nr1*	AGT CCA GCG TCT GGT TTG AG	TTC TCT GCC TTG GAC TCA CG
*Nr2a*	TGA TGA ACC GCA CTG ACC CTA	GGA AGA ACG TGG ATG TCG GA
*Nr2b*	GGG TTA CAA CCG GTG CCT A	CTT TGC CGA TGG TGA AAG AT
*Glur1*	CAA GTT TTC CCG TTG ACA CAT C	CGG CTG TAT CCA AGA CTC TCT G
*Egr1*	TAT GAG CAC CTG ACC ACA GAG	GCT GGG ATA ACT CGT CTC CA
*Bdnf*	CCT TAC TAT GGT TAT TTC ATA CTT CGG TT	TCA GCC AGT GAT GTC GTC GTC
*c-fos*	CGG GTT TCA ACG CCG ACT A	TGG CAC TAG AGA CGG ACA GAT

### Tissue Processing for Biochemical Analysis

Hippocampal CA fields were dissected as described above and cortices where dissected and pealed of the corpus callosum. Hippocampal CA tissues from WT and N1cKO mice were either fractionated to obtain the synaptosomal membrane fraction (Hou et al., 2008; *n* = 3 bilateral CA fields per fractionation) or homogenized using non-ionic NP-40 buffer (*n* = 2 bilateral CA fields per condition). Cortical tissue from Reln^−/+^ and Reln^+/+^ was dissected and fractionated to obtain the soluble (S2) and synaptic membrane fraction (P2; *n* = 2–3 bilateral CA fields per fractionation). Cortical tissue from WT mice was processed to obtain whole cell lysate using non-ionic buffer (*n* = 1 bilateral cortices per condition). Proteins’ concentrations from all preparations were determined using the BCA method (Roth, Germany).

### Co-Immunoprecipitation Assays and Western Blot Analysis

Immunoprecipitations using specific antibodies were performed on 1.2 mg of protein from either whole cell lysates or synaptosomal fractions depending on the application. The various homogenates were incubated for 1.5 h with 10 μg of primary antibodies or with 10 μg of Goat serum (PAA, Austria). Protein A/G Magnetic beads (#88802, Thermo Fisher, USA) were then added to the samples, which were incubated for another 1.5 h. Next, the beads were washed three times (0.1% Triton, 50 mM Tris-HCL pH 7.5, 300 mM NaCl). The fourth wash was performed using the washing buffer containing 0.2% SDS, and the fifth wash with PBS containing 0.1% Triton. The beads were eluted using 50 μl of 2 × Laemmli Buffer (Carl Roth, Germany). Proteins were separated using standard electrophoresis and western blot techniques. Proteins transferred to a Nitrocellulose membrane (Membrane Solutions, Germany) and were probed with primary antibodies, and Infrared dye-conjugated secondary antibodies (LiCOR, Germany). An Infrared scanner (LiCOR, Germany) was used to visualize the signal. Densitometric analysis to quantitate the intensity of individual protein bands was done using ImageJ software (NIH, USA). Values were normalized against the loading control b-actin and averaged among experiments.

### LTP Recordings and Analysis

Transverse hippocampal slices (350 μm-thick) were prepared from 7–9 week-old C57Bl/6J and N1cKO mice of either sex. Brains were cut in ice-cold, oxygenated solution containing (in mM): 105 sucrose, 65 NaCl, 25 NaHCO_3_, 2.5 KCl, 1.25 NaH_2_PO_4_, 7 MgCl_2_, 0.5 CaCl_2_, 25 glucose, 1.7 L (+)-ascorbic acid. Slices were allowed to recover at 35°C for 1 h in artificial CFS (ACSF) containing (in mM): 130 NaCl, 25 NaHCO_3_, 2.5 KCl, 1.25 NaH_2_PO_4_, 1.2 MgCl_2_, 2 CaCl_2_, 25 glucose, supplemented with 1.7 L (+)-ascorbic acid, 2 Na-pyruvate and 3 myo-inositol. In the recording chamber, slices were constantly superfused with ACFS at near-physiological temperature (30–32°C) supplemented with (in mM): 1.7 L (+)-ascorbic acid, 0.05 picrotoxin and 0.001 glycine. Field EPSPs (fEPSPs) were recorded in CA1 stratum radium through a borosilicate micropipette filled with ACSF and elicited by stimulation of Schaffer collaterals (100 μs duration, 0.05 Hz) with a tungsten concentric microelectrode (World Precision Instruments). Signals were acquired through a Digidata 1320 digitizer, amplified through a Multiclamp 700B amplifier, sampled at 4 kHz and filtered between 1 Hz and 1 kHz using Clampex10 (Molecular Devices, USA). Stimulus intensities were adjusted to elicit ~50% of the maximal response, which typically contained population spikes. After at least 20 min of stable baseline fEPSPs, LTP was induced using a theta-burst stimulation (TBS) protocol, which consisted of five trains of four pulse bursts at 200 Hz separated by 200 ms, repeated six times every 10 s. To evaluate Reelin’s effects on LTP, slices were superfused with ~5 nM Reelin or control medium for 10 min prior to TBS (Trotter et al., 2013). LTP levels were calculated as % change of the fEPSP initial slopes during min 51th to 60th after TBS as compared to the last 10 min of baseline. Statistical comparison between groups was performed with unpaired Student’s *t*-test on log-transformed values of LTP levels.

### Whole-Cell Recordings

Acute transverse hippocampal slices (300 μm thick) were prepared from 8–12 week-old N1cKO and WT mice in ice-cold solution containing (mM): 248 Sucrose, 2.5 KCl, 26 NaHCO3, 1.25 NaH2PO4, 2 CaCl2, 1 MgCl2 and 10 D-glucose. After 1 h incubation at 34°C, they were kept at room temperature in extracellular solution (ACSF) containing (mM): 125 NaCl, 2.5 KCl, 26 NaHCO3, 1.25 NaH2PO4, 2 CaCl2, 1 MgCl2, 10 D-glucose, 0.01 glycine. For whole-cell recordings, slices were placed under a microscope in a submersion-type recording chamber constantly perfused with oxygenated ACSF supplemented with 0.1 mM picrotoxin. Whole-cell recordings were performed on visually identified CA1 pyramidal neurons with 3–4 MΩ borosilicate glass pipettes filled with (mM): 127 Cs-gluconate, 6 MgCl2, 10 Hepes, 0.2 EGTA, 10 Na phosphocreatine, 2 MgATP, 0.2 Na2GTP, and 2.5 QX314. Recordings were done with a Multiclamp 700B amplifier (Molecular Devices, USA), low-pass filtered at 3 KHz, sampled at 10 KHz with a Digidata 1320 A/D interface (Molecular Devices), and acquired with pClamp 9 software package (Molecular devices). Access resistance (Ra) was monitored and cells with Ra above 30 MΩ or changing by more than 20% during the recording were discarded. A period of >10 min was observed after obtaining the whole-cell configuration before starting measurements of AMPAR- and NMDAR-mediated currents. EPSC were evoked every 20 s by monopolar stimulation with a glass pipette filled with ACSF placed in the stratum radiatum of CA1. AMPA currents were measured at −70 mV after ensuring that evoked responses were stable. Then, the holding potential was progressively brought to +40 mV and 20 μM CNQX was added to the superfusion to isolate NMDAR-mediated EPSCs. NMDA currents were measured >5 min after reaching +40 mV. CNQX blocked EPSCs recorded at −70 mV while AP5 blocked those recorded at +40 mV (in the presence of CNQX), indicating that these responses are mediated specifically by AMPAR and NMDAR, respectively.

### Measurements of NMDA/AMPA Ratio

Data were analyzed with pClamp 9 software (Molecular Devices, USA). Between 10–15 baseline-subtracted waveforms were averaged to measure AMPA and NMDA currents as described before (Gonzalez-Burgos et al., 2008). The NMDA/AMPA ratio was calculated from the area under ESPC waveforms at −70 mV and +40 mV, representing the NMDA and AMPA charge transfers, respectively. A paired Student’s *t*-test was used to compare NMDA/AMPA ratios between genotypes.

### Statistical Analysis

Quantification of the immunogold particles was performed on the length of the pre- and post-synaptic membrane. Colocalization analysis of dendritic puncta was performed on fixed primary neurons as well as on paraffin sections using the ImageJ Coloc 2 analysis (uhnresearch.ca/wcif). NMDAR1 or F-actin puncta on a single confocal slice of 0.2 μm were chosen as regions of interest (ROIs). Colocalization of Notch1/ApoER2 and Notch1/Dab1 pixels was performed on the ROIs. A total of 50 puncta from three different experiments were analyzed per staining. Colocalization of Notch1/NMDAR1 pixel was performed on ROIs selected on NMDAR1 puncta on distal regions in CA3 and CA1 on single optical slices of 0.2 μ from three animals. An average of 500 puncta per region was analyzed. Transcript and protein values were normalized to housekeeping genes and the loading controls, β-actin or GAPDH, respectively. All data were compiled and analyzed using Excel and the Real Statistic Add-in (Dr. Charles Zaiontz). Student’s *t*-test was used in all pairwise analysis for statistical comparisons.

## Results

### Notch1 Colocalizes Postsynaptically with Reelin Signaling Components

**Figure 1:**
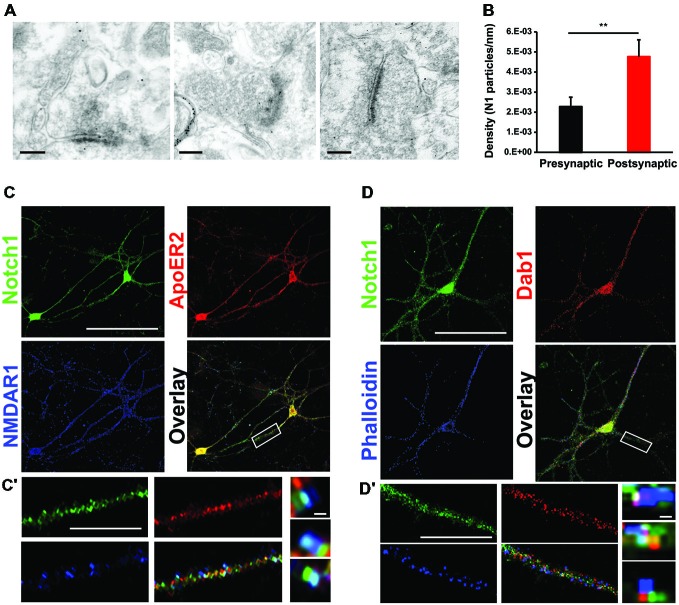
**Notch1 colocalizes postsynaptically with Reelin signaling components. (A)** Representative IEM images from hippocampal slices using an antibody specific for Notch1 show that gold particles are localized at postsynaptic as well as presynaptic membrane terminals. **(B)** Bar graph summarizing the counting of gold particles on the length of presynaptic and postsynaptic membranes indicates that the majority of the Notch1 gold particles are localized postsynaptically (5*10^−3^ ± 0.8*10^−3^ vs. 2*10^−3^ ± 0.4*10^−3^, *n* = 3 mice; Student’s *t*-test, *p* < 0.01). **(C)** Fluorescent immunolabeling on 14 days primary neuronal WT cultures shows colocalization of Notch1 and ApoER2 in soma and processes of pyramidal neurons. Both Notch1 and ApoER2 are similarly co-expressed in NMDAR1 positive puncta (*R* = 0.92 ± 0.03 and *R* = 0.86 ± 0.08, respectively; Student’s *t*-test, *p* = 0.23). **(C^′^)** Close up of a dendrite displaying Notch1, ApoER2 and NMDAR1 expression and “zoom in” captions of puncta showing clustering of the three receptors in teal. **(D)** Fluorescent immunolabeling on primary neuronal cultures shows that Notch1 and Dab1 localize in the same pyramidal neuron’s soma and processes labeled by phalloidin (*R* = 0.87 ± 0.06 and *R* = 0.74 ± 0.07 respectively; Student’s *t*-test, *p* = 0.5). **(D^′^)** Close up of a dendrite with Notch1, Dab1 and F-actin labeling and “zoom in” captions of dendritic puncta showing clustering of Notch1, Dab1 and F-actin in teal. ^**^p < 0.01. Error bars are SEM and scale bar in **(A)** is 200 nm for all IEM panels, in **(C,D)** 50 μm and in **(C^′^,D^′^)** 10 μm and 500 nm in zoom in captions.

**Figure 2:**
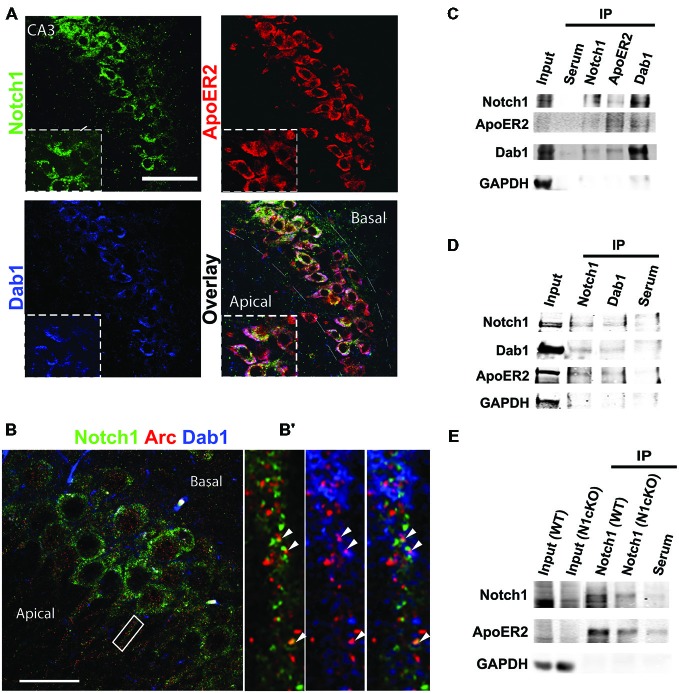
**Notch1, ApoER2 and Dab1 colocalize in hippocampal neurons. (A)** Representative image of fluorescent immunolabeling shows that Notch1 is localized in a subset of CA3 neurons, co-expressing Dab1 and ApoER2 (magnified insert). ApoER2 is highly expressed in all CA3 neurons. **(B)** Fluorescent immunolabeling shows the distribution of Notch1 and Dab1 as compared to Arc/Arg3.1 in CA3 neurons. **(B^′^)** Close up of a dendrite showing colocalization of the three proteins (white arrowheads). **(C)** Western blot analysis on immunoprecipitated samples from whole hippocampal lysate shows that Notch1 displays a strong interaction with Dab1 whereas Notch1 and ApoER2 interaction appears weaker in the preparation (*n* = 4 independent experiments). **(D)** Immunolabeling on immunoprecipitated samples from whole cortical lysates show that Notch1 and Dab1 interact. ApoER2 is bound to both Notch1 and Dab1 (*n* = 3 independent experiments). **(E)** Co-IP from synaptosomal membrane fractions reveals a stronger interaction between Notch1 and ApoER2, which is reduced in the N1cKO (*n* = 3 independent pulldowns). In **(C–E)** GAPDH is used as a loading control for the inputs and to detect contamination in the IP samples. Scale bar in **(A)** is 50 μm and **(B)** 25 μm.

In the adult mouse brain, among the Notch receptors, Notch1 appears to be the dominant Notch signaling receptor (Sestan et al., 1999; Redmond et al., 2000; Stump et al., 2002). Previous work from our laboratory has indicated that Notch1 and the Jagged1 ligand are localized at synapses (Alberi et al., 2011). To establish the localization of the Notch1 receptor at Schaffer collateral CA3-CA1 synapses, we have conducted gold immuno-electron-microscopy (IEM) on hippocampal cryosections using a specific antibody against the cytoplasmic tail of Notch1. We observe that Notch1, as visualized by gold particles, is localized post and presynaptically (Figure 1A). Countings of the gold particles along the synaptic membranes length on 74 synapses from three independent labelings (*n* = 3 mice) indicate that the majority of the gold particles are concentrated postsynaptically as compared to the presynaptic terminal (*p* < 0.01, Student’s *t*-test; Figure 1B). Analysis of postsynaptic puncta in hippocampal neuronal cultures confirms that Notch1 is strongly localized in puncta, which are positive for the principal NMDAR subunit, NMDAR1 (NR1), (*R* = 0.92 ± 0.03) or the spines’-enriched actin isoform, F-actin (phalloidin) (*R* = 0.87 ± 0.06; Figures 1C,D). In addition, at the NR1 positive puncta, where Notch1 is expressed, the Reelin receptor, ApoER2 can be found at a comparable frequency as Notch1 (*R* = 0.86 ± 0.08; Figure 1C). Similarly, the secondary messenger of Reelin transduction, Disabled 1 (Dab1), localizes in Notch1 positive F-actin puncta (*R* = 0.74 ± 0.07; Figure 1D). Furthermore, Notch1 and ApoER2 show comparable colocalization coefficients to Notch1 and Dab1 (*R* = 0.75 ± 0.06 vs. *R* = 0.71 ± 0.05; *p* = 0.5). The colocalization of the Notch1 receptor with Reelin signaling components, ApoER2 and Dab1, was confirmed by fluorescent immunolabeling on hippocampal slices. ApoER2 appears uniformly distributed in all pyramidal neurons of the CA3 field (Figure 2A). On the other hand, Notch1 is expressed in a neuronal subset (Figure 2A, insert). Interestingly, the same neurons expressing Notch1 are also positive for Dab1, suggesting that Notch1 and Dab1 hallmark specific neuronal ensembles (Figure 2A, insert). Since Notch1 displays a non-canonical interaction with Arc/Arg3.1, which facilitates Notch1 activity in hippocampal networks (Alberi et al., 2011), we investigated whether Notch1/Dab1 and Arc/Arg3.1 are part of the same molecular complex (Figure 2B). We observe that the three proteins colocalize in dendritic puncta (Figure 2B^′^, arrowheads). To confirm the physical interaction between Notch1 and Reelin signaling components in the hippocampus, we performed coimmunoprecipitation (co-IP) on whole cell lysates using specific antibodies against Notch1, ApoER2 or Dab1 (Figure 2C). Notch1 interacts with Dab1, in line with the *in vitro* and *on slice* immunolabeling. As expected, Dab1 and ApoER2 physically interact (Figure 2C), whereas the interaction between Notch1 and ApoER2 appears weaker (Figure 2C). The interaction between Notch1, Dab1 and ApoER2 was confirmed by co-IP on cortical lysate (Figure 2D). Since both Notch1 and ApoER2 are transmembrane receptors colocalizing in postsynaptic puncta on hippocampal neurons, the interaction between ApoER2 and Notch1 was further validated using Notch1 co-IPs from hippocampal membrane fractions. We observe that the physical interaction in this preparation is greater (Figure 2E) as compared to the pulldown using the same antibody on hippocampal cell lysates (Figure 2C). The physical contact is reduced in synaptosomal fractions obtained from conditional KO mice lacking Notch1 in hippocampal CA fields (N1cKO; Alberi et al., 2011). The residual signal in the N1cKO lanes may originate from other neurons than pyramidal neurons or from glia, where Notch1 is still present. Yet, this experiment validates the specificity of Notch1 and ApoER2 binding (Figure 2E). The physical interaction between the Notch1 and the Reelin receptor, ApoER2, at the synapse and its secondary messenger, Dab1, in whole cell lysates hints at a functional crosstalk between the two pathways in neurons.

### Loss of Notch1 Affects Reelin Signaling

**Figure 3:**
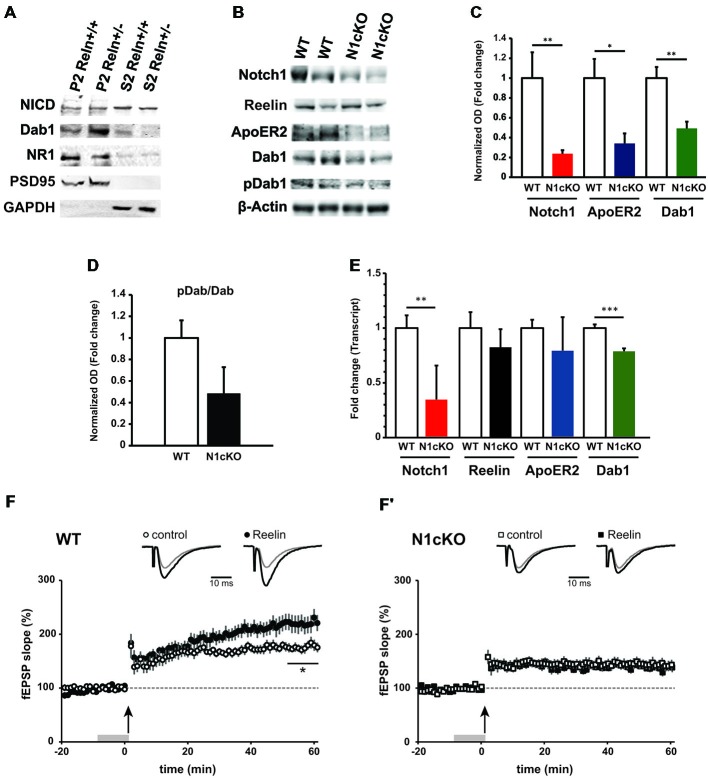
**Loss of Notch1 interferes with the Reelin cascade and Reelin-mediated synaptic potentiation. (A)** Western blot analysis on synaptosomal (P2) and cytosolic (S2) fractions from the cortex of Reelin heterozygous mice (Reln^+/−^) and wildtype controls (Reln^+/+^) shows that total NICD levels are not affected in both compartments. Moreover, Dab1 levels are increased and NMDAR1 expression is unchanged in the haploinsufficient (Reln^+/−^) mice (*n* = 3 independent fractionations). **(B)** Representative immunoblot on whole hippocampal lysates show a reduction in Notch1, ApoER2 and Dab1 expression in the N1cKO as compared to WT. Reelin and Dab1 phosphorylation appear unchanged (*n* = 3 independent experiments). **(C)** Bar graph summarizing the optical density analysis of Notch1, ApoER2 and Dab1 bands normalized to β-actin. In N1cKO hippocampi, a 76.2% reduction is observed in Notch1 (*n* = 6 mice per genotype; Student’s *t*-test, *p* < 0.01), a 65.9% reduction in ApoER2 (Student’s *t*-test, *p* < 0.05) and 50.7% reduction in Dab1 (*n* = 5 mice per genotype; Student’s *t*-test, *p* < 0.01) as compared to WT controls. **(D)** Dab1 phosphorylation is not significantly reduced in N1cKO mice (*n* = 5 mice per genotype; Student’s *t*-test, *p* = 0.09) as compared to control littermates. **(E)** qPCR analysis shows the transcript expression of Notch1, Reelin, ApoER2 and Dab1 in wildtype and N1cKO hippocampi. Dab1 in N1cKO mice is decreased by 20% as compared to WT (*n* = 4 per genotype; Student’s *t*-test, *p* < 0.001). Reelin and ApoER2 transcripts do not change. Notch1 transcript is reduced by 65% in N1cKO mice (*n* = 4 per genotype; Student’s *t*-test, *p* < 0.01). **(F,F^′^)** Field EPSPs (fEPSPs) were elicited in CA1 stratum radiatum by Schaffer collateral stimulation. LTP was induced with TBS. Upper traces represent average fEPSP recorded during baseline (gray) and 50 min after TBS (black). **(F)** In WT, superfusion of Reelin-conditioned medium (Rln) 10 min prior to TBS (gray bar) induces and promotes LTP (•, *n* = 9 slices, 6 mice) as compared to control medium (ctr; °, *n* = 9 slices, 7 mice; Student’s *t*-test, *p* < 0.05). **(F^′^)** In N1cKO mice, LTP is impaired in both Rln and control conditions (□, *n* = 9 slices, 4 mice; ▪, *n* = 9 slices, 5 mice; *p* = 0.63). *p = < 0.05, Data are averages ± SEM.

Previous evidence has indicated that Reelin signaling positively regulates Notch processing in the developing forebrain through Dab1-mediated interaction (Hashimoto-Torii et al., 2008; Sibbe et al., 2009; Keilani et al., 2012). To determine whether Notch and Reelin signaling cooperate similarly at the synapse, we investigated Notch1 expression and processing in WT (Reln^+/+^) littermate controls as compared to the Reelin heterozygous mice (Reln^+/−^), which display normal brain anatomy (Qiu et al., 2006). We observe that in synaptosomal preparation from the Reln^+/−^ cortices the total NICD, as readout of Notch cleavage, is indistinguishable from the Reln^+/+^ control mice (Figure 3A). Whereas, as expected, Dab1 is increased due to reduced turnover (Bock et al., 2004) and NR1 expression is unchanged (Qiu et al., 2006; Figure 3A). Thus, in the adult brain, Notch signaling does not appear to be downstream of Reelin signaling. Yet, given the significant interaction between Notch1 and Reelin signaling components in hippocampal neurons (Figures 1, 1, 2) and the striking similarities in memory deficit between the loss of function for Reelin (Beffert et al., 2005; Trotter et al., 2013) and Notch1 (Costa et al., 2003; Wang et al., 2004; Alberi et al., 2011), we hypothesized that Notch1 and Reelin may functionally synergize to mediate synaptic plasticity. To address this possibility, we took advantage of the N1cKO mouse line (Alberi et al., 2011). Immunoblot analysis on whole hippocampal lysate reveals that, although Reelin levels appear unaffected between the two genotypes, ApoER2 and Dab1 levels are critically reduced in the N1cKOs as compared to WTs (Figures 3B,C). On the other hand, Dab1 phosphorylation is unchanged (Figure 3D), suggesting that Src kinase activity is intact (Arnaud et al., 2003; Benhayon et al., 2003). Transcript analysis indicates that there is a small but significant decrease in Dab1 transcript as compared to WT, whereas Reelin and ApoER2 transcripts remain unchanged between genotypes (Figures 3C,E). Notch1 protein and transcript reduction confirms the significant deletion of Notch1 in the CA fields. Interestingly, Dab1 has been identified as a canonical target of Notch1 (Li et al., 2012). To further assess whether, in the absence of Notch1, Reelin signaling is affected, we investigated the effect of Reelin bath application on hippocampal plasticity. It has been previously shown that Reelin binding to ApoER2 facilitates the opening of NMDARs inducing synaptic potentiation (Weeber et al., 2002). As expected, Reelin bath application increases LTP under TBS in WT hippocampal slices as compared to control medium (Figure 3F). On the other hand, in the Notch1cKO, TBS fails to induce LTP. Moreover, the Reelin-mediated potentiation is absent in the N1cKO (Figure 3F^′^). Thus, we hypothesized that besides the impairment in Reelin-mediated plasticity in the N1cKOs, NMDARs function (Tsien et al., 1996) may be compromised in absence of Notch1.

### Notch1 Regulates NMDA Transmission

**Figure 4:**
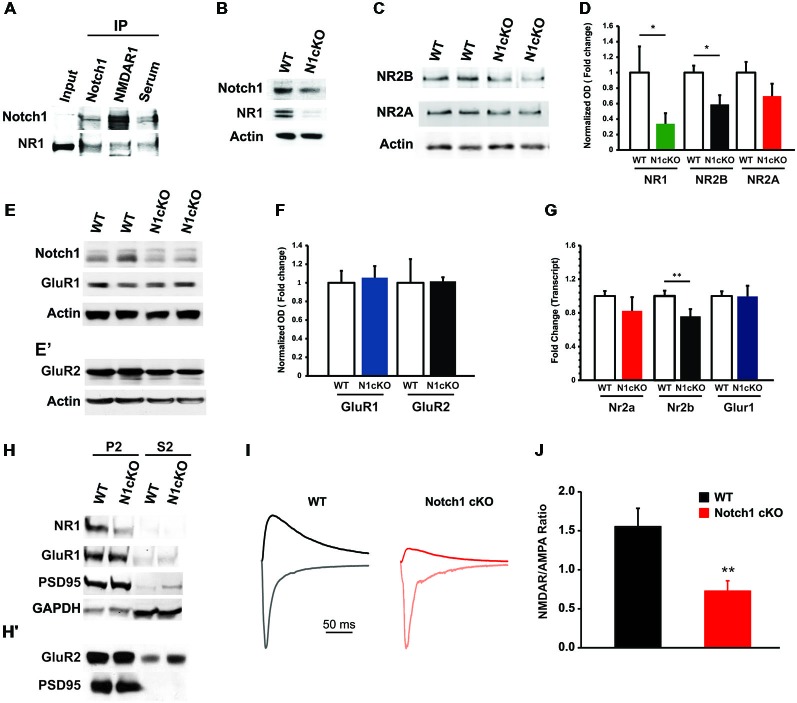
**Notch1 affects NMDAR composition and conductance. (A)** Western blot on co-IPed synaptosomal fraction shows that Notch1 and NMDAR1 physically interact (*n* = 3 independent experiments). **(B)** Representative western blot on WT and N1cKO whole hippocampal lysates indicates that NR1 is visibly reduced in the N1cKO (*n* = 5 independent experiments). **(C)** Immunoblot on whole hippocampal lysate indicates a reduction in NR2B expression in the N1cKO as compared to WT. NR2A does not appear affected (*n* = 4 independent experiments). **(D)** Bar graph summarizing the changes in protein expression of NR1, NR2B and NR2A normalized against β-actin. NR1 expression is reduced by 66.1% in the N1cKO mice (*n* = 6 mice per genotype; Student’s *t*-test, *p* < 0.05), NR2B is decreased by 41.2% in the KOs (*n* = 5 mice per genotype; Student’s *t*-test, *p* < 0.05), whereas NR2A has a 30.3% reduction in KO mice but is not significantly different from WT (*n* = 5 mice per genotype; Student’s *t*-test, *p* = 0.20; *n* = 4 animals per genotype). Immunoblot analysis on synaptosomal fraction shows that AMPA receptor subunits, **(E)** GluR1 and **(E^′^)** GluR2, are not affected in N1cKO mice as compared to the WT littermates (*n* = 3–6 independent experiments). **(F)** Bar graph indicates no difference in the expression of GluR1 (*n* = 5 mice per genotype; Student’s *t*-test, *p* = 0.16) and GluR2 (*n* = 5 mice per genotype; Student’s *t*-test, *p* = 0.95) between WT and N1cKO mice. **(G)** Bar graph showing the fold changes in transcript expression of NR1 (Nr1), NR2B (Nr2b), NR2A (Nr2a) and GluR1 (Glur1). Only mRNA levels of NR1 (0.62 ± 0.02 vs. 1 ± 0.13, *n* = 3–4 animals per genotype; Student’s *t*-test, *p* < 0.05) and NR2B (0.75 ± 0.09 vs. 1 ± 0.06, *n* = 4 animals per genotype; Student’s *t*-test, *p* < 0.01) are decreased in KO hippocampi as compared to WT. **(H,H^′^)** Immunoblots on synaptosomal (P2) and soluble (S2) fractions. **(H)** NR1 is reduced, whereas GluR1 and PSD95 tagging at the synapse appear unaffected. GAPDH is used as a positive control for the cytosolic fractions. **(H^′^)** GluR2 expression is not changed at the synaptic level (*n* = 4 independent experiments). **(I)** NMDAR- and AMPAR-mediated evoked responses in CA1 pyramidal neurons. Scaled sample current traces recorded at −70 mV (gray traces) and +40 mV in the presence of CNQX (black traces) from WT and N1cKO mice. For easier comparison of NMDAR/AMPAR ratio, the current at +40 mV was scaled to the peak current at −70 mV of the same recording. **(J)** Bar graph shows that the mean NMDAR/AMPAR is significantly decreased in N1cKO mice (*n* = 13–15 neurons per genotype; Student’s *t*-test, *p* < 0.006). Data are averages ± SEM, *p < 0.05 and ^**^p < 0.01.

High frequency stimulation (HFS; Wang et al., 2004; Alberi et al., 2011) and TBS fail to induce LTP in the N1cKO. It is established that LTP is triggered by charge transfer through NMDARs (Berberich et al., 2007) and potentiation is maintained through synaptic tagging of α-amino-3-hydroxy-5-methyl-4-isoxazolepropionic acid receptors (AMPARs; Kessels and Malinow, 2009). In addition, Reelin signaling regulates NMDARs composition and synaptic maturation (Qiu and Weeber, 2007). We therefore hypothesized that loss of Notch interferes either with NMDAR and/or AMPAR function or with downstream messengers of glutamatergic transmission. We first validated the physical colocalization between Notch1 and NR1, which is the constitutive subunit of NMDAR (Tsien et al., 1996). We performed co-IPs on synaptosomal membrane fractions using an antibody against the intracellular portion of Notch1 and extracellular portion of NR1. We observed that Notch1 and NR1 co-precipitate (Figure 4A), supporting a functional interaction between the two membrane receptors, also revealed by the colocalization analysis in the primary hippocampal cultures (Figure 1) and hippocampal sections (Supplementary Figure 2). To further explore such interaction, we carried out immunoblotting on hippocampal cell lysate. Transcript and protein levels of the NR1 subunit appear severely decreased in N1cKO as compared to WT controls (Figures 4B,D,G). Inspection of the NMDAR2 (NR2) subunits reveals a 41% reduction in NMDAR2B (NR2B) protein levels in the N1cKO hippocampi but no significant change in NMDAR2A (NR2A) between genotypes (Figures 4C,D). Since Reelin regulates NR2A synaptic tagging (Qiu and Weeber, 2007) and NR2B mobilization (Groc et al., 2007) without any effect on NR1 nor NR2A or NR2B expression, the NMDAR composition defect in the N1cKO depends on Notch1 loss only. To further understand whether the lack of Notch1 also affects AMPAR, we investigated the composition of AMPAR. AMPA receptors are found as GluR1/2 and GluR2/3 heterodimers (Wenthold et al., 1996). In the N1cKO hippocampi, levels of ionotropic AMPA receptor subunits, GluR1 and GluR2 appear unchanged as compared to WT (Figures 4E,F). Transcript analysis confirms that mRNA levels of the NR2B (Nr2b) are mildly (25%) but significantly reduced in N1cKO hippocampi (Figure 4G), whereas neither Nr2a nor Glur1 are significantly affected (Figure 4G). The reduction in synaptic NR1 was confirmed on synaptosomal membrane fractions and appears to be independent of PSD95 anchoring (Figure 4H). As expected from the analysis of total AMPA receptor levels, GluR1 and GluR2 positioning at the synapse is not altered in these mutants (Figures 4H,H^′^). Based on the significant alteration in NMDAR composition and expression, we investigated NMDA and AMPA conductance using whole-cell voltage-clamp recordings from pyramidal CA1 neurons. The NMDAR/AMPAR ratio is decreased by 50% in N1cKO mice as compared to WT controls, whereas AMPA currents appear unaffected (Figures 4I,J). Thus, loss of Notch1 directly affects NMDAR composition and function.

### Notch1 is Upstream of CREB Activity

**Figure 5:**
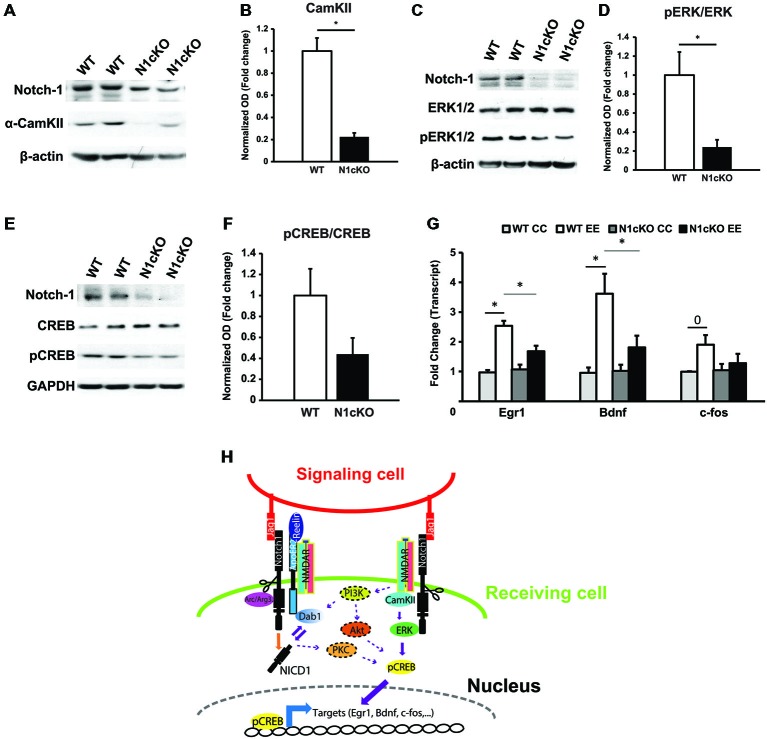
**Notch1 affects CREB phosphorylation and transcription. (A)** Immunoblot on whole hippocampal lysates shows a substantial reduction in CamKII in the hippocampi of N1cKO as compared to WT. β-actin is used as a loading control (*n* = 3 independent experiments). **(B)** Quantification of CamKII protein expression between N1cKO and WT normalized to β-actin shows a reduction by 78% in the N1cKO hippocampi (*n* = 6 animals per genotype; Student’s *t*-test, *p* < 0.05). **(C)** Western blot on hippocampal lysates shows that ERK1/2 phosphorylation (pERK1/2) in N1cKO is reduced as compared to WT hippocampi. β-actin is used as a loading control (*n* = 3 independent experiments). **(D)** Quantification of ERK1/2 phosphorylation in N1cKO as compared to WT indicates a 76% decrease in the KO samples (*n* = 6 mice per genotype; Student’s *t*-test, *p* < 0.05). **(E)** Immunoblot on whole hippocampal lysates shows a reduction in CREB phosphorylation in N1cKO as compared to WT (*n* = 3 independent experiments).** (F)** Bar graph shows a near to significance 56% reduction in CREB phosphorylation in the N1cKO hippocampi as compared to WT (*n* = 4 animals per genotype; Student’s *t*-test, *p* = 0.069). **(G)** Graph summarizing transcripts expression of CREB targets, Egr1, Bdnf and *c*-fos, in hippocampi from cage control (CC) and environmental exposed (EE) WT and N1cKO mice (*n* = 3–4 mice per genotype; Student’s *t*-test, *p* < 0.05; Black bars compare WT CC with WT EE; gray bars compare WT EE with N1cKO EE). **(H)** Model of the proposed Notch1 non-canonical signaling regulations of Reelin, NMDA and CREB pathways described in this study. Elements putatively connecting Notch1/Reelin signaling to Notch1/Glutamaterigic signaling are dotted and are based on the existing literature. Data are averages ± SEM and *=*p* < 0.05; 0 signifies *p* = 0.5.

The increase in calcium (Ca^2+^) influx through NMDAR determines the activation and the localization of the calcium calmodulin kinase II (CamKII) to the postsynaptic terminal (Coultrap and Bayer, 2012). In addition, NR2B binds to CamKII with high affinity leading to its persistent activation (Lisman et al., 2012). To simulate a rise in synaptic potentiation *in vivo* and to investigate secondary messengers of NMDAR transmission, we exposed WT and N1cKO mice to novel environmental exploration (EE) for 5 min. During this time, regardless of their reported memory impairment (Alberi et al., 2011), N1cKOs display normal exploratory activity. On the other hand, immunoblot on hippocampal lysates showed a drastic reduction (75%) in CamKII levels in the mutants, 90 min after exploration (Figures 5A,B). Furthermore, ERK phosphorylation, which occurs through CamKII and Ras activation (Wang et al., 2009) is critically reduced in the N1cKO hippocampi as compared to WT controls (Figures 5C,D). ERK activates CREB through phosphorylation (Impey et al., 1998). This event is fundamental for CREB- dependent transcription (Davis et al., 2000). We observed that in absence of Notch1, CREB phosphorylation is affected nearly to significance as compared to WT (Figures 5E,F). As a consequence of reduced CREB phosphorylation, we hypothesized that CREB transcription would be affected in the mutants. Thus, we analyzed transcript levels of Egr1, Bdnf and c-fos, which are all validated targets of CREB (Impey et al., 2004), in cage control (CC) and environmentally exposed (EE) animals. After novel environmental exposure, we observed a significant induction of Egr1 and Bdnf in WT mice. However, N1cKOs fail to display the same increase as compared to WTs. On the other hand, the increase in c-fos transcripts between WT CC and WT EE appears near to significance and c-fos levels appear unchanged between genotypes (Figure 5G). On the whole, it appears that the loss of Notch1 can affect a cascade of secondary messengers associated with plasticity and spatial learning.

## Discussion

In this paper, we shed light on relevant mechanisms underlying Notch-dependent function in neurons. This work is crucial to understand the implications of Notch1 in memory and learning.

### Notch1 and Reelin Signaling Crosstalk at the Synapse

We have previously shown that Notch1 signaling is induced in hippocampal ensembles by increased neuronal activity and through the interaction with the early immediate gene, Arc/Arg3.1 (Alberi et al., 2011). This was the first example of a non-canonical interaction, which is essential to warrant activity-dependent Notch signaling in neurons. Yet, Notch1 and Arc/Arg3.1 mutant mice display profoundly different plasticity defects (Alberi et al., 2011; Plath et al., 2006), suggesting that along with the interaction between Notch1 and Arc/Arg3.1, other molecular crosstalks determine Notch1 function. Interestingly, Reelin signaling shares many functions with Notch1 in the adult brain: (i) spine maturation; (ii) synaptic plasticity (Weeber et al., 2002); and (iii) memory formation (Beffert et al., 2005; Qiu et al., 2006; Trotter et al., 2013). As for Notch alterations, Reelin signaling imbalances are observed in AD mouse models and have also been reported in human AD (Kocherhans et al., 2010). Furthermore, previous studies have indicated a critical interaction between Notch1 and Reelin signaling in cortical migration and neuronal maturation (Hashimoto-Torii et al., 2008; Sibbe et al., 2009). The latter works using the Reelin loss of function model, Reln^−/−^, provide evidence for Notch1 as a downstream regulator of Reelin activity. However, here, we show that Reelin haploinsufficiency does not affect Notch1 activity, despite compromising hippocampal plasticity and contextual fear memory (Qiu et al., 2006). On the other hand, we find that Notch1 interacts with Reelin signaling components (Figures 1, 1, 2) and can influence Reelin-dependent processes at the synapse (Figure 3). The synaptic function of Notch1 is further supported by the gold IEM on hippocampal slices and correlates well with the localization of ApoER2 (Beffert et al., 2005) and Dab1 (Trotter et al., 2013) in postsynaptic puncta (Figure 1). Loss of Notch1 in the hippocampus profoundly alters ApoER2 and Dab1 proteins levels without any effect on Reelin (Figure 3). This is in line with the expression of Reelin in hippocampal interneurons and not pyramidal neurons, where Notch1 is deleted. Furthermore, Dab1 phosphorylation is reduced but not to a significant level, suggesting that residual Reelin signaling may account for Src-mediated phosphorylation of Dab1 (Arnaud et al., 2003; Benhayon et al., 2003). This may further reduce the levels of Dab1 based on the proteasomal activation achieved through phosphorylation (Bock et al., 2004). Indeed, the transcript analysis indicates that Dab1 is mildly but significantly (20%) reduced in the N1cKO hippocampus, whereas ApoER2 transcripts remain unchanged. Nevertheless, based on the existence of different splicing variants in neurons (Koch et al., 2002), it remains possible that an imbalance in ApoER2 isoforms expression in the N1cKO may have remained undetected using a primer set common to the 12 variants. The impairment of Reelin signaling in the N1cKO is further corroborated by the evidence that Reelin stimulation, which potentiates LTP (Weeber et al., 2002) in the WT hippocampi, fails to improve LTP in absence of Notch1 in a manner similar to the Dab1cKO mouse line (Trotter et al., 2013; Figure 3). Reelin facilitates synaptic potentiation and it is thought to be one of the fundamental cell signaling mechanisms in learning and memory function (Herz and Chen, 2006). Our data positions Notch1 as a key regulator of Reelin signaling and Reelin-mediated synaptic potentiation. This may explain why the Notch1 haploinsufficient and N1cKO mouse models (Costa et al., 2003; Wang et al., 2004; Alberi et al., 2011) phenocopy the plasticity and memory deficits observed in the ApoER2KOs (Weeber et al., 2002; Beffert et al., 2005) and Dab1cKO (Trotter et al., 2013).

### Notch1 Regulates NMDAR Activity

In the N1cKO, the induction of LTP using either HFS (Alberi et al., 2011) or TBS, with or without Reelin application, is severely impaired. This suggests a deficiency in membrane conductance and Ca^2+^ permeability in absence of Notch1. NR1 is ubiquitous to all NMDAR and possesses high Ca^2+^ permeability. We demonstrate that Notch1 strongly interacts with the NR1 subunit at the synapse (Figure 4). This interaction is not achieved through ApoER2 since this receptor preferentially binds to the NR2A subunit (Beffert et al., 2005). Furthermore, loss of Notch1 severely impacts the expression of NR1 and NR2B, whereas NR2A remains unaffected. Despite a developmental switch from NR2B to NR2A in the mature hippocampus (Monyer et al., 1994), the majority of NMDARs populating the adult synapse are NR1/NR2A/NR2B triheteromeric (Rauner and Köhr, 2011). As such, the strong reduction in NR1 and NR2B in the N1cKO interferes with NMDAR availability and function (Figure 4). On the other hand, AMPA receptors do not appear to be influenced by the loss of Notch1. The specificity of NMDAR dysfunction in the N1cKO is further supported by the loss of NR1 at the synapse and the strong reduction in NMDA currents leaving AMPA currents unaffected (Figure 4). These effects share strong similarity with the loss of γ-secretase function in the presenilin 1 (PS1) and presenilin 2 (PS2) KOs (PScDKO; Saura et al., 2004) and the ApoER2Δex19 mouse line (Beffert et al., 2005). Notch1 and ApoER2 are both direct targets of the γ-secretase complex. Moreover, loss of PS1/PS2 may interfere with Notch1 availability by shunting a positive feedback loop induced by canonical Notch signaling, as previously shown in *Drosophila* (Ahimou et al., 2004; Del Monte et al., 2007). Thus, the present study indicates Notch1 as the relevant γ-secretase substrate with synaptic plasticity function by distinct, yet converging mechanisms impinging on Reelin signaling and NMDAR transduction. In support of the Notch1-dependent regulation of NMDAR function, N1cKO hippocampi display both LTP and LTD reduction (Alberi et al., 2011), which underlie NMDAR function (Collingridge et al., 1983; Dudek and Bear, 1992). Based on the sensible reduction of NR1 and NR2B in the N1cKO, we observe not only an absence in LTP induction (Tsien et al., 1996; Barria and Malinow, 2005), but also an inhibition of LTD (Dudek and Bear, 1992; Massey et al., 2004) comparable to the loss of NR1 and/or NR2B in the hippocampus (Barria and Malinow, 2005). Furthermore, it has to be noted that the loss of Notch1 affects more dramatically protein levels of NR1, NR2B as well as ApoER2 and Dab1 as compared to transcript levels (Figures 3–4). This raises the interesting possibility that Notch1 may be involved in proteins’ turnover/stability by yet unknown mechanisms.

### Notch1 Regulates ERK and CREB Activation

Loss of Notch1, besides affecting NMDAR composition, dramatically reduces CamKII expression in a comparable manner to the PScDKO mice (Saura et al., 2004; Figure 5). CamKII activity is induced by Ca^2+^ binding and requires association to NR2B (Coultrap and Bayer, 2012). These events regulate LTP (Barria and Malinow, 2005; Halt et al., 2012) as well as learning and memory (Silva et al., 1992). Interestingly, all these deficits can be observed in the N1cKOs (Alberi et al., 2011). At present, it is unclear why the levels of CamKII are reduced in the N1cKO. However, it is plausible that the reduction in NR2B levels prevents the synaptic positioning of CamKII (Lisman et al., 2012), causing default degradation of the untagged proteins (Tsai, 2014). Furthermore, loss of Notch1 affects ERK phosphorylation (Figure 5). This may be explained by concomitant factors occurring in absence of Notch1: (i) failure in CamKII activity (Schmitt et al., 2005); (ii) loss of Dab1 function (Trotter et al., 2013); and (iii) interference with Akt activity (Marathe et al., 2015). As a result, diminished ERK leads to a nearly significant decrease in CREB phosphorylation at Ser142 (Davis et al., 2000; Impey et al., 2004) and disruption in CREB-dependent Egr1 and Bdnf transcription. On the other hand, c-fos appears unchanged (Figure 5). This discrepancy may be explained by the timing of observation, 90 min after having completed a novel EE. Indeed, c-fos activation occurs within 15 min after sensory experience and decays to basal level within 1 h (Guzowski et al., 2001). On the other hand, after contextual conditioning, Egr1 and Bdnf display prolonged expression lasting a couple of hours (Hall et al., 2000). Moreover, the latter genes appear to be instrumental for LTP maintenance and memory formation, whereas the role of c-fos in memory remains so far elusive (Alberini, 2009). This emphasizes the recruitment of Notch1 in conditions of increased synaptic activity (Alberi et al., 2011) and points to a critical role of Notch1 in regulating CREB-targets, which are essential for the establishment of memories. Interestingly, at least two other works, in the fruit fly and mouse, have indicated a positive interaction between Notch and CREB signaling (Saura et al., 2004; Zhang et al., 2013). In particular, the study in *Drosophila* has shown that, after learning, Notch1 follows an ultradian oscillation pattern, which affects CREB phosphorylation (Zhang et al., 2013). A time-lapse analysis of Notch1 and CREB activity in the hippocampus of rodents following learning would establish whether such circadian oscillations occur also in mammals.

In summary, this work provides the first mechanistic evidence for a role of Notch in synaptic plasticity. Interestingly, all the crosstalks described in this paper are non-canonical interactions involving ApoER2 and NMDAR functions (Figure 5H). At present, it remains unclear how these two functional complexes are connected or whether they are part of the same synaptic super-complex. Some putative interactions may be inferred from previous works (Figure 5H). However, further investigations are needed to finally resolve Notch1 non-canonical interactions at the synapse as opposed to Notch1 canonical/transcriptional signaling. Results from this paper further support the role of Notch signaling in memory processing and provide the foundation for understanding how impairment in Notch signaling, as observed in AD, may contribute to dementia.

## Conflict of Interest Statement

The authors declare that the research was conducted in the absence of any commercial or financial relationships that could be construed as a potential conflict of interest.
