# Supplementary figures and images for: Notch1 Regulates Hippocampal Plasticity Through Interaction with the Reelin Pathway, Glutamatergic Transmission and CREB Signaling
Source: Front Cell Neurosci. 2015 Nov 26;9:447. doi: 10.3389/fncel.2015.00447 (PMC4659909; doi:10.3389/fncel.2015.00447)

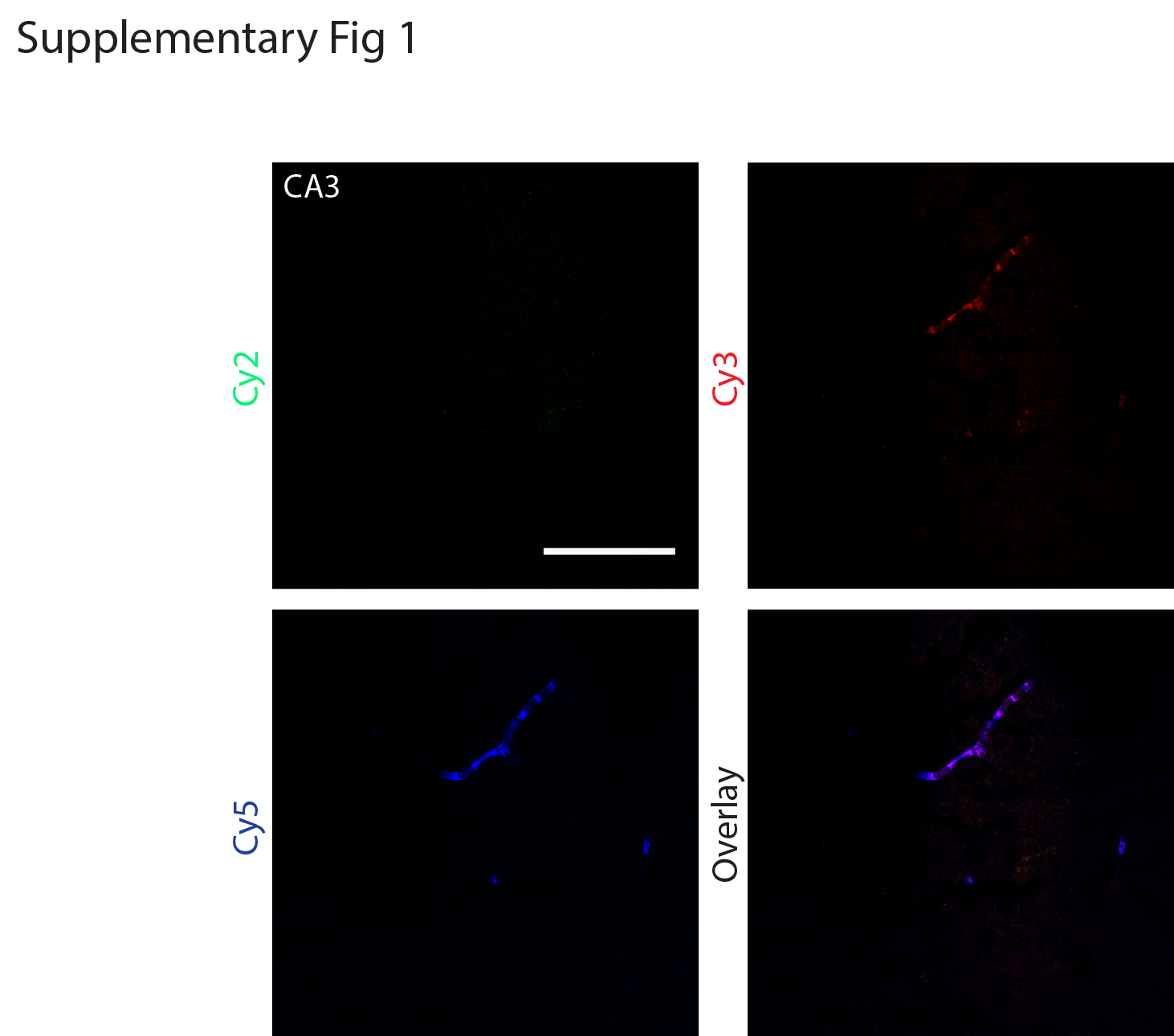

Supplement: Supplementary file 1 [file Image_1.tif]

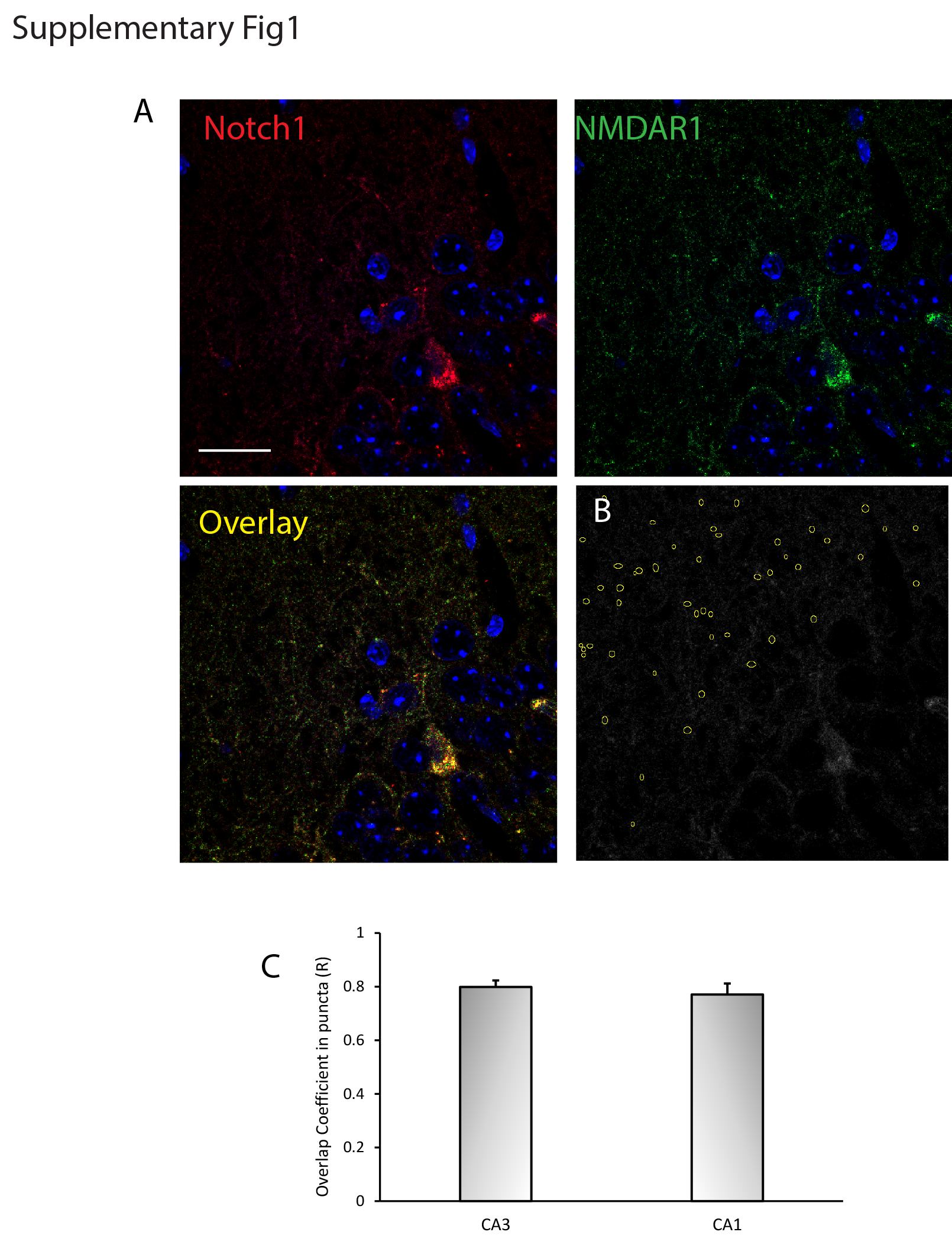

Supplement: Supplementary file 2 [file Image_2.tif]
