# Supplementary material for: Notch1 Regulates Hippocampal Plasticity Through Interaction with the Reelin Pathway, Glutamatergic Transmission and CREB Signaling
Source: Front Cell Neurosci. 2015 Nov 26;9:447. doi: 10.3389/fncel.2015.00447 (PMC4659909; doi:10.3389/fncel.2015.00447)
Supplement: Supplementary file 3 [file Presentation_1.pdf]

Notch1 regulates hippocampal plasticity through interaction with the Reelin pathway, glutamatergic transmission and CREB signaling

Emanuele Brai[1], Swananda Marathe[1], Simone Astori[2], Naila Ben Fredj[1], Elisabeth Perry[3], Christophe Lamy[1], Alessandra Scotti[4] and Lavinia Alberi[1,5*]

[1] Institute of Anatomy, Department of Medicine, University of Fribourg, Fribourg, Switzerland.

[2] Department of Fundamental Neurosciences, University of Lausanne, Lausanne, Switzerland.

[3] Histology Core Laboratory, Georgia Health Sciences University, Department of Cellular Biology and Anatomy, Augusta, Georgia, USA.

[4] Institute of Anatomy, Faculty of Medicine, University of Bern, Bern, Switzerland.

[5] Unit of Pathology, Department of Medicine, University of Fribourg, Fribourg, Switzerland.

Supplementary Material

Figure 1. Routine secondary antibody immunolabeling for background subtraction. Representative image of hippocampal CA field immunolabeling using the secondary antibodies against Goat (Cy2), Rabbit (Cy3) and Mouse (Cy5). Image is used for background subtraction on the immunolabeling of Notch1 (Cy2), ApoER2 (Cy3) and Dab1 (Cy5) in Figure 2A. Scale bar in A is 50 μm.

Figure 2. Notch1 colocalizes with NMDAR1 in CA1 and CA3 distal dendritic puncta. A) Fluorescent immunolabeling using Notch1 (red) and NMDAR1 (green) specific antibodies on a 200 nm optical slice form 6 μm paraffin section of CA3 hippocampal fields. **B)** Capture of the selected ROIs in the apical region of the CA3 neurons in the optical slice of 200 nm in A. **C**) Bar graph summarizing the extent of colocalization of Notch1 and NMDAR1 in selected ROIs in CA3 and CA1 distal regions (n=3 independent experiments) (R=0.79±0.02 and R=0.77±0.04). Data are averages ± SEM. Scale bar in A is 25 μm.
